# Utilization of Municipal Solid Waste Ash in Concrete Blends in Israel Part B: Combustion in a Semi-Industrial Incinerator

**DOI:** 10.3390/ma19132686

**Published:** 2026-06-23

**Authors:** Sarit Nov, Shay Barak, Haim Cohen, Yaniv Knop

**Affiliations:** 1 Department of Chemical Sciences, Ariel University, Ariel 40700, Israel; hcohen@ariel.ac.il; 2 Department of Chemical Engineering, Ariel University, Ariel 40700, Israel; 3 Department of Chemistry, Ben Gurion University of the Negev, Beer Sheva 84105, Israel; 4 Department of Civil Engineering, Ariel University, Ariel 40700, Israel

**Keywords:** municipal solid waste ash (MSW ash), waste-to-energy (WtE), circular economy, waste valorization, concrete

## Abstract

This study (Part B) examines the potential utilization of municipal solid waste (MSW) ash, produced in a semi-industrial incinerator in Israel, as a partial substitute for cement and natural sand in industrial concrete mixtures. The ash was produced at the temperature range 600–850 °C, and the ash was characterized using XRD and SEM to determine its mineralogical composition and morphology. The results indicate that ash composition is dominated by calcium-rich phases, with hatrurite (Ca_3_SiO_5_) representing approximately 51–66 wt.% of the identified crystalline phases, along with calcite, MgO, and silica phases. The ash consists of irregular, porous particles with a broad distribution. Concrete performance was evaluated in both fresh and hardened states. In terms of fresh concrete properties, it is observed that concrete containing ash showed improved workability, better workability retention, and better concrete density compared to concrete without ash. In terms of hardened concrete properties, the use of MSW ash as a partial sand replacement preserved the mechanical performance of the concrete, with compressive strength remaining within approximately 2% of the reference mixture. These findings suggest that semi-industrially produced MSW ash is more suitable as a fine aggregate replacement than as a supplementary cementitious material and represents a promising route for reducing landfill disposal and promoting circular economy practices in the construction industry.

## 1. Introduction

Concrete manufacturing in Israel plays a central role in infrastructure development. Still, it is associated with significant environmental burdens, primarily due to the energy-intensive production of cement and the extensive extraction of fine aggregates, such as sand and gravel [1,2]. Cement production is a major contributor to greenhouse gas emissions, releasing large quantities of carbon dioxide (CO_2_) through the calcination of limestone and the combustion of fossil fuels [3]. Simultaneously, aggregate extraction leads to habitat destruction, soil erosion, and the depletion of non-renewable natural resources, thereby exacerbating environmental degradation [4]. These challenges necessitate sustainable alternatives that reduce the environmental footprint of concrete without compromising performance [5]. Partial replacement of Portland cement with supplementary cementitious materials (SCMs) is a well-established strategy for reducing clinker consumption and associated CO_2_ emissions [6]. Fly ash, a byproduct of coal combustion in thermal power plants, has long been recognized for its pozzolanic properties, which contribute to improved concrete strength, durability, and workability, while reducing the overall cement demand [7]. However, the global energy transition away from coal-fired power generation has led to a decline in fly ash availability, prompting the construction industry to explore alternative SCMs [8]. Among these, municipal solid waste (MSW) ash, derived from the incineration of MSW (which is expanding as a promising mode for MSW treatment), has gained attention as a promising candidate due to its chemical composition and potential environmental benefits [9]. MSW incineration typically generates two main solid residues, bottom ash and fly ash, which differ significantly in particle size, chemical composition, and environmental risk profile. Bottom ash is the main ash product, generally coarser and less enriched in volatile heavy metals, whereas fly ash often contains higher concentrations of soluble salts and potentially hazardous elements. These differences strongly influence their suitability for incorporation into cement-based systems. MSW ash typically contains silica, alumina, calcium oxide, and other mineral phases that may contribute either to pozzolanic reactions or to filler and microstructural effects, depending on physicochemical characteristics [10]. Particle size distribution and fineness significantly influence reactivity and filler performance, as finer particles enhance packing density and surface-driven reactions. The mineralogical composition of incineration ash is highly dependent on combustion conditions and thermal history, which may significantly affect its performance in cementitious systems [11]. However, reactivity cannot be inferred solely from bulk oxide composition. The presence of crystalline calcium phases, limited amorphous silica, and variable combustion conditions may significantly affect its hydraulic or pozzolanic performance. Therefore, each ash source requires detailed mineralogical and microstructural characterization before its incorporation into cement-based systems.

Beyond material performance, MSW ash valorization reduces landfill disposal, conserves virgin resources, and supports circular economy principles [12]. This approach aligns with circular economy principles, emphasizing waste minimization, resource efficiency, and material reuse, which are increasingly prioritized in national and international sustainability agendas.

Cement substitution with industrial byproducts can substantially reduce concrete-related CO_2_ emissions, with reductions exceeding 50% at high replacement levels [10,13]. Diverting MSW ash from landfills further decreases disposal volumes and associated long-term environmental liabilities and the appreciable cost of the overall incineration process. Environmental safety considerations, particularly heavy metal content and leaching behavior, remain critical for regulatory acceptance. Economically, MSW ash may reduce raw material costs for concrete producers. Simultaneously, it enables waste management facilities to convert residual waste into a value-added resource, improving the economic feasibility of waste-to-resource strategies [14] and reducing the need for long-term storage of incineration ash, which can significantly increase the waste management costs of ashes produced by the incineration of MSW, which appreciably increases incineration costs.

The incorporation of municipal waste ash into concrete mixtures has been reported to provide both performance improvements and reductions, depending on material characteristics. However, the variability in reported performance indicates that ash properties are strongly source- and process-dependent, reinforcing the need for site-specific evaluation. Experimental studies [15,16] as well as recent reviews on solid waste utilization in construction materials [17] show that, when adequately processed and properly graded, MSW ash may enhance compressive and tensile strength. However, contradictory results have also been reported, particularly when the ash exhibits low amorphous content or high concentrations of crystalline calcium compounds, leading to limited pozzolanic reactivity and, in some cases, strength reduction. Moreover, the ash can improve durability by reducing permeability, enhancing resistance to sulfate attack, and mitigating alkali–silica reactions [18]. However, limited data are available for MSW ash produced under semi-industrial combustion conditions representative of emerging waste-to-energy (WtE) systems. These gaps underscore the need for evaluation under realistic operational scales.

In Israel, the potential for utilizing municipal waste ash in concrete is particularly relevant given the country’s evolving waste management landscape. The government is starting to promote WtE technologies, including incineration, as part of its strategy to reduce landfill dependence and recover energy from MSW [19]. As incineration capacity expands, MSW ash generation is expected to increase significantly, necessitating sustainable management solutions. Local utilization in concrete production may reduce transportation-related impacts and enhance overall resource efficiency. This transition presents an opportunity to integrate waste management with construction material innovation within a circular economy framework [20].

Based on Part A of this study [21], which characterized MSW ash generated under laboratory-scale combustion conditions, the present work (Part B) focuses on ash produced in a semi-industrial incinerator to reflect realistic operational conditions better. The working hypothesis is that MSW ash generated at a semi-industrial scale may, depending on its mineralogical characteristics, be used either as a supplementary cementitious material or as a fine aggregate substitute.

Accordingly, this study systematically evaluates the mineralogical properties of the ash. It assesses its effects on the performance of fresh and hardened concrete when used as a partial replacement for cement or natural sand.

## 2. Experiment

### 2.1. Materials

**Figure 1:**
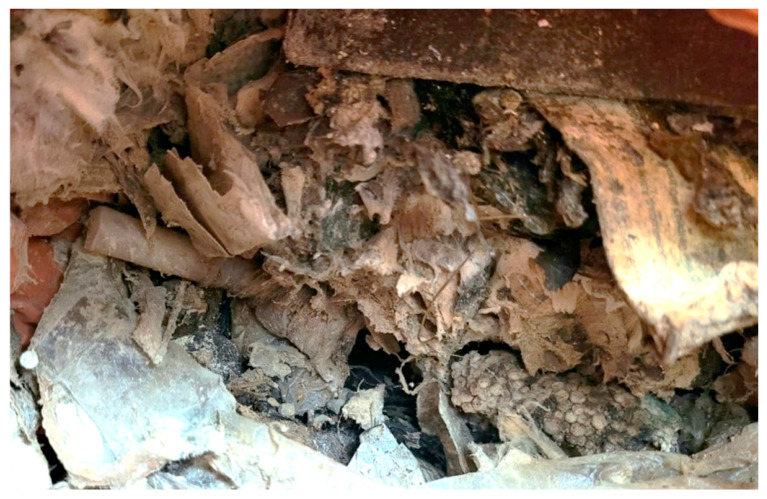
A sample of MSW intended for landfill at the Dudaim site [21].

The Dudaim Reclamation Center supplied municipal solid waste (MSW), as seen in Figure 1 [21]. The MSW used in this study was pre-sorted to remove metallic components, aluminum cans, and large plastic fractions before incineration. As reported in Part A of this research [21], the moisture content of the incoming waste stream varied between 35 and 70 wt.%, reflecting the heterogeneous nature of municipal solid waste.

The cement used in this study was commercial Portland cement CEM I 52.5 N (Nesher, Ramla, Israel). The cement had a Blaine fineness of 3800 cm^2^/g and a density of 3.12 g/cm^3^. The chemical composition was dominated by CaO (60.0 wt.%) and SiO_2_ (19.7 wt.%), with additional amounts of Al_2_O_3_ (6.0 wt.%), Fe_2_O_3_ (3.4 wt.%), MgO (1.1 wt.%), and SO_3_ (2.8 wt.%). The total loss on ignition (LOI) was 3.1 wt.%, indicating a low content of volatile components and confirming the cement’s suitability for concrete production.

### 2.2. Analysis and Methods

SEM analysis: The ash samples were mounted on aluminum stubs using conductive aluminum tape and analyzed using an Ultra-High Resolution Maia 3 Field Emission Scanning Electron Microscope (FE-SEM, Tescan Maia 3, Brno, Czech Republic) at the Electronic Microscopy Lab, Technology and Engineering Unit, Ariel University. The observations were performed using a secondary electron (SE) detector at an accelerating voltage of 10 KeV. SEM images were collected at different magnifications to evaluate particle morphology, surface texture, and particle size distribution. No conductive coating was applied prior to analysis.

XRD analysis: The ash samples were analyzed using an X’pert Pro X-Ray Diffractometer by PANalytical Company (Malvern, UK) at the Surface Laboratory of Ariel University. XRD patterns were collected using a Cu Kα radiation source (λ = 1.5406 Å) in θ–2θ geometry over a 2θ range of 5–70°, with a step size of 0.01° and a counting time of 1 s per step (scan rate 0.6° min^−1^). Measurements were performed at room temperature using fixed divergence and receiving slits.

Concrete tests: The MSW ash was tested by mixing CEM I 52.5 N cement and measuring the compressive strength in accordance with SI 118 standard [22]. The compressive and flexural strengths were tested using a control press after the samples were cured in water at a temperature of 21 ± 30 °C for 7 days, and after 7 days, the samples were cured in an open space until the day of pressing.

### 2.3. The Semi-Industrial Incinerator

**Figure 2:**
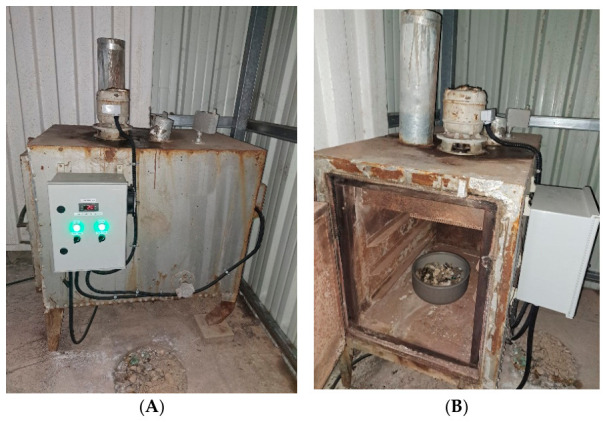
(**A**) The semi-industrial incinerator, where MSW incineration was conducted. (**B**) A sample of MSW inside the incinerator before the incineration process.

Because it was not feasible to incinerate significant quantities of waste in the laboratory furnace in Part A [19], it was not possible to obtain sufficient ash quantities for experiments to prepare concrete mixtures using ash from municipal waste incineration as a partial replacement for cement and aggregates. For this purpose, a large furnace (Figure 2) was upgraded to a semi-industrial incinerator, enabling the incineration of larger quantities of waste and producing greater quantities of MSW bottom ash to be used as a replacement for cement and aggregates in concrete mixtures.

### 2.4. Concrete Mixtures Preparation

The MSW ash was used as a partial substitute for cement and for natural sand in concrete. The concrete containing a mixture of pure cement and MSW ash was tested to produce industrial concrete mixtures. Industrial concrete mixtures were prepared and tested according to SI 118 standard to evaluate the effect of MSW ash as one of the concrete component mixtures on the concrete properties. The cement used is CEM I 52.5 N.

The blended cement pastes were prepared and tested according to EN-197 standard [23] to evaluate the effect of municipal waste ash on the concrete strengths as a partial cement replacement.

In order to test the ability to partially replace cement with ash, cement pastes were tested in accordance with EN 197 in the mixture compositions shown in Table 1.

To study the effect of MSW ash, 35 g. of cement was replaced with 35 g of MSW ash. The mixtures were prepared with a constant water-to-(cement + ash) ratio.

The properties of the hardened concrete were measured; compressive strengths were determined after 7 days and 28 days after casting. The compressive strength value reported is the average of four samples.

The mix design of the concrete is presented in Table 2.

## 3. Results and Discussion

### 3.1. Incineration of MSW in a Semi-Industrial Incinerator

The incineration of MSW was conducted in a semi-industrial incinerator following a controlled thermal profile to ensure complete combustion. In each experiment, a defined quantity of MSW (as detailed in Table 3) was introduced into the furnace and heated. After approximately 20 min, the temperature reached ~250 °C, at which point substantial smoke evolution began, indicating the release of moisture and volatile compounds and the onset of combustion. This smoking phase continued as the temperature increased to ~450 °C, marking the transition to sustained combustion. The combustion process then proceeded within the temperature range of approximately 450–850 °C, with smoke emission diminishing as burning stabilized. The system was maintained for two hours to ensure complete combustion of the organic fraction, during which the average temperature reached ~850 °C. Following the process, only bottom ash and residual glass remained in the ceramic vessel, and the glass fragments were subsequently removed.

The remaining ash from the three experiments was combined and mixed to obtain a homogeneous sample, and samples were taken for XRD and SEM analyses.

### 3.2. Untreated MSW Ash

**Figure 3:**
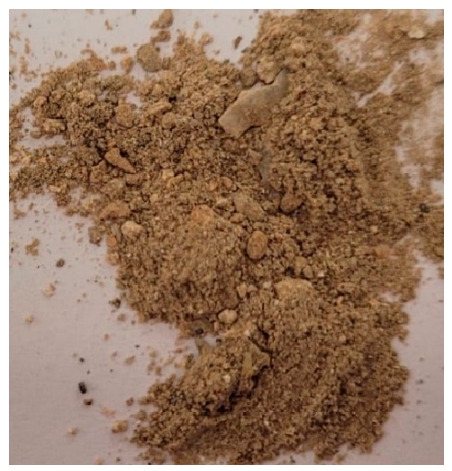
Untreated MSW ash.

**Figure 4:**
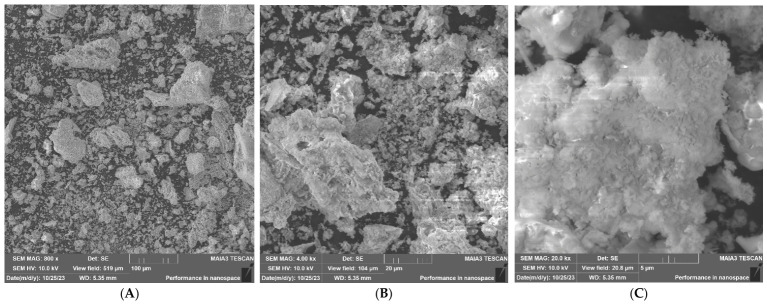
SEM images of MSW ash at various magnifications. (**A**) Magnification of 100 μm, (**B**) magnification of 20 μm, and (**C**) magnification of 5 μm.

The MSW ash consisted of brown particles. A photograph of untreated MSW ash is presented in Figure 3. XRD analyses determined the mineralogical composition, as presented in Table 4, and magnified SEM images of the ash are presented in Figure 4.

The ash composition in both samples is dominated by hatrurite (Ca_3_SiO_5_), accounting for 65.7% in Sample 1 and 51% in Sample 2. Additional calcium-based minerals, including calcite (CaCO_3_), magnesium oxide (MgO), and portlandite (Ca(OH)_2_), are also present in notable amounts. Quartz (SiO_2_) was detected in Sample 1 (4%), whereas Sample 2 contains a relatively higher amount (15%). Overall, the results indicate that the ash is rich in the calcium- and silica-containing phases typical of cement-related mineral systems.

Differences between Samples 1 and 2 are primarily attributed to the heterogeneous nature of MSW and to minor variations in combustion conditions. Despite these differences, both samples exhibited similar mineralogical trends characterized by the predominance of calcium-rich phases, indicating overall consistency in ash composition.

Based on the mineralogical phases identified via XRD analysis (Table 4), the theoretical oxide composition of the untreated MSW ash samples was calculated by stoichiometric conversion. This analysis assumes the complete conversion of the quantified crystalline phases into their corresponding oxide components. The resulting distribution of the major oxides for both Sample 1 and Sample 2 is presented in Table 5.

The calculated oxide composition confirms that the ash is dominated by CaO (51–61 wt.%), whereas the silica content is considerably lower (21–28 wt.%). This composition differs from typical pozzolanic materials such as coal fly ash, which are generally richer in reactive amorphous silica and alumina. The predominance of calcium-rich crystalline phases identified by XRD is consistent with the limited cementitious reactivity observed in the mechanical performance tests.

Comparison with previously published studies indicates that the composition of MSW incineration ash can vary considerably depending on waste composition and combustion conditions. Previous investigations reported the presence of quartz, aluminosilicate phases, calcium-bearing minerals, and glassy components in different proportions [9,16,20]. In the present study, the ash was dominated by hatrurite (Ca_3_SiO_5_), together with calcite, portlandite, and MgO. The calculated oxide composition revealed a CaO content of approximately 51–61 wt.% and a SiO_2_ content of 21–28 wt.%, indicating a calcium-rich material. The high calcium content is consistent with the formation of calcium-bearing crystalline phases during high-temperature combustion processes [9,10].

The SEM images reveal that the MSW ash consists of irregular, heterogeneous particles with a wide particle size distribution. At lower magnification (A), the ash appears as agglomerates of coarse and fine particles. Higher magnifications (B–C) show rough, porous surfaces and clusters of smaller crystalline fragments attached to larger particles. This morphology suggests a mechanically fragmented material with a high surface area, which may influence packing behavior and interactions within cement-based mixtures.

Based on SEM observations at different magnifications (5–100 µm scale range), the MSW ash exhibits a broad particle size distribution. The material contains fine particles in the sub-micron range, medium-sized particles of approximately 1–10 µm, and larger agglomerates reaching tens of micrometers (up to ~50–100 µm). This wide distribution supports improved packing density in cementitious mixtures. However, it should be noted that this estimation is based on SEM imaging and does not represent a full quantitative particle size distribution.

To complement the SEM observations, particle size distribution (PSD) of the MSW ash was determined by laser diffraction (Malvern Mastersizer). The results indicated a broad particle size distribution with Dv(10), Dv(50), and Dv(90) values of 1.4, 15.7, and 228 µm, respectively. The volume-weighted mean particle size D[4,3] was 73.9 µm, while the surface-area-weighted mean particle size D[3,2] was 3.07 µm. The calculated specific surface area was 1956 m^2^/kg. The wide particle size distribution is consistent with the heterogeneous morphology observed in the SEM images and may contribute to improved particle packing in concrete mixtures.

### 3.3. Concrete Mixtures

**Figure 5:**
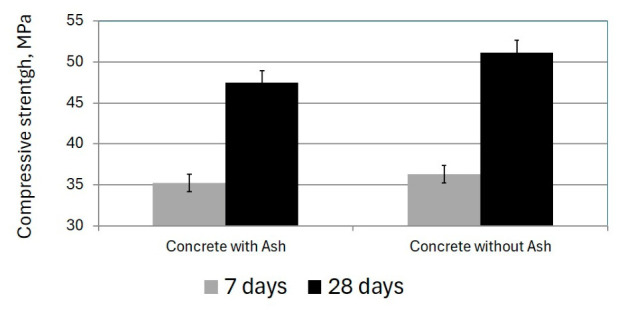
Compressive strength after 7 and 28 days from casting using MSW ash.

Figure 5 presents the compressive strengths after 7 and 28 days from casting. As shown, there is a reduction in the strength both after 7 days and after 28 days as compared to the samples that did not contain MSW ash. From this, it can be concluded that the strength of the concrete was reduced (~10%) due to the reduction in the amount of cement in the concrete and that the ash does not contain reactive components that can partially replace the cement.

After testing the ability of ash to partially replace cement, industrial concrete mixes were tested to examine the use of ash to replace natural sand and the effect of ash on the properties of fresh concrete.

**Figure 6:**
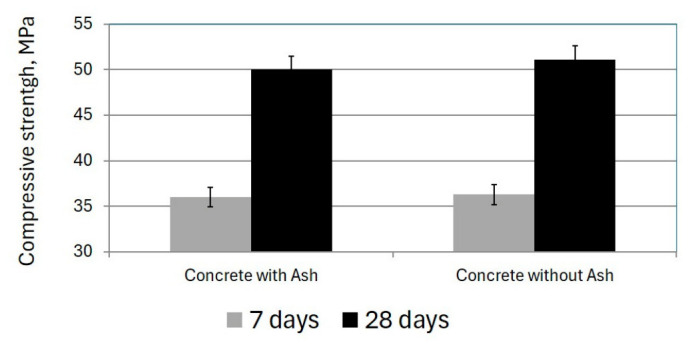
Compressive strength after 7 and 28 days from casting using untreated MSW ash.

Figure 6 presents the compressive strengths after 7 days and after 28 days in a mixture containing 23 kg of ash as a partial replacement of sand. The compressive strengths were tested according to SI 26 part 4.1 [22]. As shown in the figure, the compressive strengths did not change significantly (~2%) after either 7 days or 28 days between the concrete mixture with the ash and the concrete without the ash. From this, it can be concluded again that the ash is not reactive but can partially replace the use of natural sand without having a negative impact on the concrete properties of the hardened concrete. However, according to the standard, both concrete mixtures, with and without ash, are suitable as concrete type B—40.

In addition to testing the effect of ash on the strength of hardened concrete, the effect of ash in concrete on the properties of fresh concrete was examined.

**Figure 7:**
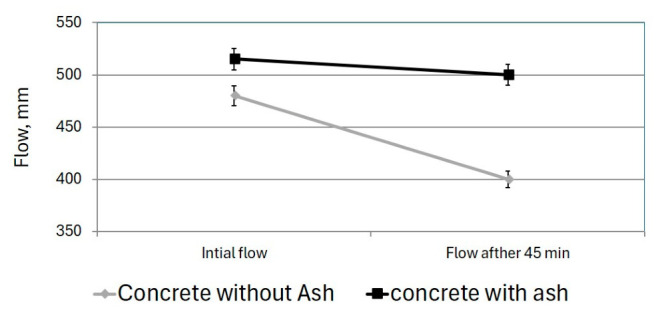
The flow after 10 and 45 min. of the concrete mixtures.

Figure 7 shows the concrete’s flowability as measured according to standard 26 part 2.4 [22]. The flowability was measured after 10 min of mixing and after 45 min to assess the mixture’s stability over time. As can be seen, the flowability of the mixture containing ash is higher and better maintained over time. The initial flow with the ash is 7% higher compared to the mixture without ash, and the flow after 45 min is 20% higher compared to the flow obtained in the concrete without ash. In addition, the flow reduction in the concrete with ash is 3% compared to 17% of concrete without the ash. Obtaining and maintaining better workability over time indicates better quality of the fresh concrete containing ash. The hypothesis for improving the properties of fresh concrete is that an increase in the packing density of the concrete mixture improves these properties. Incorporating ash at the prescribed amount improved the packing of the concrete and filled voids. Therefore, better workability was obtained despite a constant water–cement ratio.

**Figure 8:**
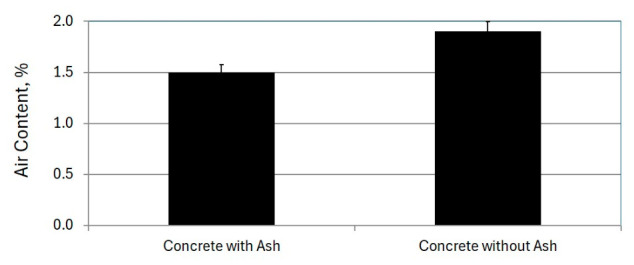
The air content of the concrete mixtures.

Another important property of the fresh concrete was the air content, which was measured according to SI 26 part 2.6 [22]. As shown in Figure 8, the air content decreased by 0.4% in the concrete mix with ash compared to the mix without ash.

Obtaining a concrete mix containing ash with a lower air content indicates a higher mix density and concrete mix cohesion achieved by using ash as a partial replacement for natural sand. The improvement in the properties of the fresh concrete in terms of workability and lower air content proves that the concrete mix grading is better and denser by using ash as a partial replacement for natural sand, as can also be seen in measuring the density of the concrete.

**Figure 9:**
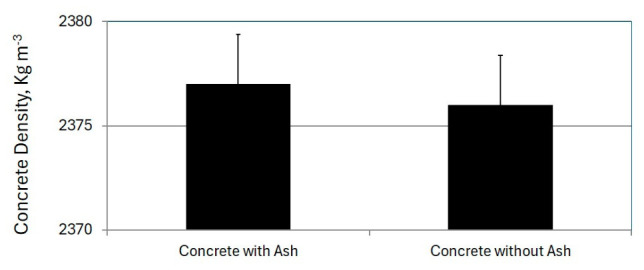
The raw bulk density of the concrete.

Figure 9 presents the bulk density of the concrete that was tested according to SI 26 part 2.5 [22] with and without ash. The bulk density of concrete with ash is 2% higher than the bulk density of concrete without ash.

As shown, replacing the natural sand improves the properties of fresh concrete, with no negative effect on the concrete’s strength. The improvement in concrete flow, reduction in air content, and the increased bulk density can be understood by using a fine powder that increases the density of the concrete and causes improved flow of the concrete and a reduction in air content.

In terms of concrete mixtures containing ash as a partial replacement for cement and as a partial replacement for natural sand, it can be concluded that there is an improvement in the properties of fresh concrete, which is expressed in a better degree of workability, maintenance of workability over time, concrete density, and denser concrete. These improvements in concrete properties indicate obtaining better, cheaper, and more sustainable concrete while producing concrete containing ash with a reduced negative impact on the environment.

## 4. Conclusions

This study evaluated the incorporation of municipal solid waste (MSW) ash, generated under semi-industrial incineration conditions, as a partial replacement for cement and natural sand in concrete mixtures. The main conclusions are as follows:

1. Practical industrial use of MSW in the concrete industry: Concrete mixtures containing MSW ash exhibited improved fresh concrete performance. The concrete containing ash obtained improved workability, better workability retention, increased density, and lower air content. These characteristics indicate that such mixtures are suitable for practical industrial application and show potential for future use.
2. Practical potential for replacing natural resources: MSW ash can be effectively used as a partial replacement for natural sand or fine aggregates without having a negative effect on the mechanical performance of hardened concrete. Although the ash showed limited pozzolanic reactivity and is therefore not suitable as a direct supplementary cementitious material, it can still be incorporated as a filler or fine aggregate component in concrete systems.
3. Environmental contribution: The utilization of MSW ash in concrete contributes to reducing environmental impacts by decreasing the demand for natural raw materials, particularly natural aggregates, and by diverting waste from landfills. This approach supports circular economy principles and promotes more sustainable construction practices.
4. Industrial relevance and future outlook: The findings demonstrate that MSW ash is particularly suitable for use in industrial concrete applications, where improvements in fresh properties and material efficiency are advantageous. Future work will focus on optimizing ash treatment methods and evaluating its potential applicability in civil and structural concrete to expand its use in broader construction contexts.

## Figures and Tables

**Table 1:** Composition of the tested mix design according to EN-197.

Material	Concrete Without Ash g	Concrete with Ash g
CEM I 52.5 N	450	415
Sand	1350	1350
Ash	-	35
Water	225	225

**Table 2:** Composition of the tested mix design.

Material	Concrete Without Ash kg m^−3^	Concrete with Ash kg m^−3^
CEM II 52.5 N	350	350
Fine Aggregate	900	900
Sand	850	827
MSW Ash	-	23
Admixtures	5.6	5.6
Water	207	207

**Table 3:** Ash data obtained from MSW incineration.

	Mass of MSW Before Burning [g]	Mass of Ash Obtained [g]	Percentage of Mass Loss [w%]	The Mass of Ash Obtained After Removing Glass Pieces [g]	Percentage of Mass Loss [w%]
1	243	73	69.90	60	75.3
2	1048	353	66.30	218	79.2
3	1250	322	74.24	200	84.0

**Table 4:** XRD analysis of untreated MSW ash.

Weight Percentage %	Sample 1	Sample 2
Hatrurite—Ca_3_(SiO_4_)O	65.7	51
Calcite—CaCO_3_	12.1	14
Magnesium Oxide—MgO	10.1	12
Portlandite—Ca(OH)_2_	8.1	8
Quartz—SiO_2_	4	-
Silicon Dioxide—SiO_2_	-	15

**Table 5:** Calculated theoretical oxide composition of untreated MSW ash samples (wt.%).

Oxide Component (wt.%)	Sample 1	Sample 2
CaO	61.3	51.5
SiO_2_	21.3	28.4
MgO	10.1	12
CO_2_	5.3	6.1
H_2_O	2	2
Total	100	100

Note: Based on the phase-to-oxide conversion, the calculated oxygen fraction in the oxide-based composition is 38.5 wt.% for Sample 1 and 40.8 wt.% for Sample 2.

## Data Availability

The original contributions presented in this study are included in the article. Further inquiries can be directed to the corresponding authors.
